# Rolando Fracture

**Published:** 2014-06-06

**Authors:** Aditya Sood, Mark S. Granick

**Affiliations:** Division of Plastic Surgery, Rutgers New Jersey Medical School, Newark, NJ

**Keywords:** Rolando fracture, Bennet fracture, thumb metacarpal fractures, base of thumb fractures, wrist fractures

**Figure:**
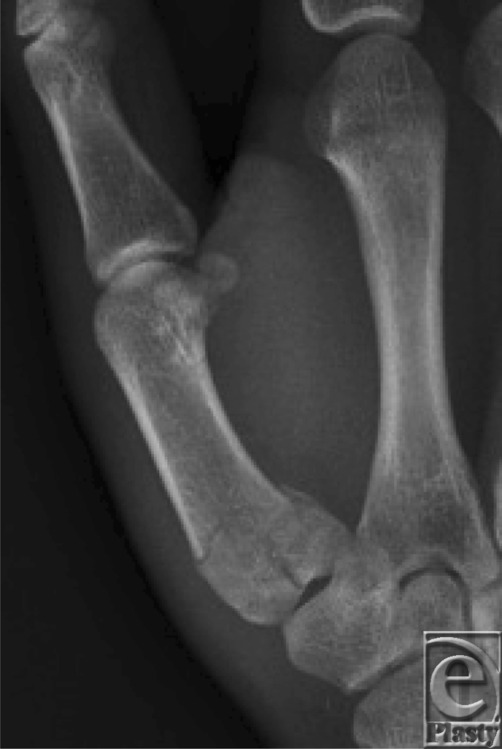


## DESCRIPTION

A 64-year-old man presented to the emergency department after fall with a chief complaint of left wrist pain. On physical examination, the patient was found to have swelling and tenderness of his left radial wrist. He denied any paresthesias, tingling, or numbness.

## QUESTIONS

1. **What are the types of intra-articular fractures at the base of the thumb?**
2. **What is the mechanism and pathoanatomy of this injury?**
3. **What are some of the treatment options?**
4. **What are some of the complications that can arise from treatment?**

## DISCUSSION

Thumb metacarpal fractures can be classified as shaft or base fractures, with base fractures further classified into extra-articular or intra-articular. Intra-articular fractures are described as either Bennet-type or Rolando-type fractures and present treatment challenges secondary to the deforming forces acting at the base of the thumb.^1^ The Bennet-type fracture is a 2-part fracture through the volar-ulnar aspect of the base of the metacarpal and is the most common variant. The Rolando-type fracture was first described in 1910 as a Y-shaped pattern with 3 major segments: the metacarpal shaft, the volar fragment, and the dorsal fragment. The term has come to mean all intra-articular thumb metacarpal base fractures with 3 or more segments, with highly comminuted fractures often placed in this category.^3^

Similar to all metacarpal base fractures, the Rolando fracture is often caused by an axial load crushing the articular surface. The base is usually split into a volar and dorsal fragment with the volar oblique ligament attached to the volar segment and the shaft pulled proximally and dorsally primarily by the abductor pollicis longus and the adductor pollicis. The patient often presents with a swollen and tender thumb base. Varus angulation of the joint may be visible. If left untreated, articular incongruity can lead to debilitating arthritis.

**Figure 1:**
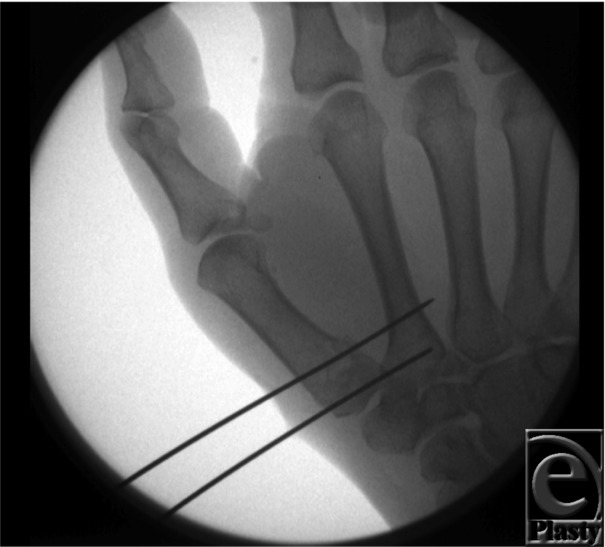
Closed reduction and percutaneous pin fixation of a Rolando-type fracture.

Treatment of a Rolando fracture can be difficult due to the comminuted nature of this fracture pattern, but surgical treatment is recommended for unstable fractures. In the presence of large volar and dorsal fragments, open reduction with internal fixation is indicated. The articular surface is reconstructed with K-wires and then secured to the metacarpal shaft using a T plate. Neurovascular damage is a major complication of this approach, and care should be taken to not damage the branches of the superficial radial nerve, the lateral antebrachial cutaneous nerve, and the radial artery.^4^ In the absence of large volar and dorsal fragments, closed reduction with external fixation can be performed.^5^^,^^6^^,^^7^ Several such techniques have been described in the literature, with closed reduction followed by percutaneous pinning with K-wires being one such method (Fig 1). To achieve reduction, the thumb is abducted, pronated, and longitudinal traction is applied. Pintract infection is a complication of this method.^8^

The Rolando fracture has been associated with a poorer prognosis than its counterpart, the Bennett fracture, largely due to its comminuted nature. Exact anatomic reduction of the articular surface may not be necessary to obtain a good functional result; however, reduction to 1 mm or less is generally thought to reduce the risk of radiographic arthritis.^8^ Studies have shown decreased palmar abduction, key pinch strength, and DASH scores in patients with Rolando fractures versus Bennett fractures.^9^

In summary, fractures of the base of the first metacarpal are particularly common. Correct diagnosis, imaging, and treatment options must be judiciously carried out to prevent debilitating joint arthritis. The aim of treatment should be exact reduction, either by the open or by the closed method.
